# Diarrhoeagenic *Escherichia coli* associated with childhood diarrhoea in Osun state, Nigeria

**DOI:** 10.1186/s12879-024-09793-0

**Published:** 2024-09-08

**Authors:** Ademola A. Olayinka, Ibukunoluwa O. Oginni-Falajiki, Iruka N. Okeke, Aaron O. Aboderin

**Affiliations:** 1 Department of Medical Microbiology and Parasitology, College of Health Sciences, Obafemi Awolowo University, Ile-Ife, Osun Nigeria; 2 Department of Pharmaceutical Microbiology, Faculty of Pharmacy, University of Ibadan, Ibadan, Nigeria; 3 Department of Medical Microbiology and Parasitology, Obafemi Awolowo University Teaching Hospitals Complex, Ile-Ife, Nigeria

**Keywords:** Antimicrobial resistance, Diarrhoea, Diarrhoeagenic *Escherichia coli*

## Abstract

**Introduction:**

Diarrhoea is a major public health concern in developing countries, usually exacerbated by poor water, sanitation and hygiene but its aetiology is under-studied, particularly away from capital cities. We identified diarrhoeagenic *Escherichia coli* (DEC) from stools collected in Ile-Ife and Ilesa, Osun state, Nigeria and determined their antibiotic resistance profiles.

**Methods:**

Stool samples from 167 children with diarrhoea and 334 controls under the age of 5 years were cultured for *Escherichia coli* and *Salmonella*. Bacterial isolates were identified biochemically and DEC were identified by PCR. Antimicrobial susceptibility testing was by modified Kirby-Bauer disc diffusion method in accordance with the CLSI guidelines. Data were analyzed using Chi-square and Fisher’s exact tests.

**Result:**

Diarrhoea infection is significantly high among children under 12 months (*p =* 0.002), caregivers without at least primary school education (*p =* 0.006), breastfeeding for under 6 months (*p*˂0.001), and caregivers who were siblings (*p =* 0.004). DEC was detected in 69(41.3%) cases but only 86(25.7%) controls (*p <* 0.001) and more commonly recovered during the wet season (*p <* 0.001). Enterotoxigenic *E. coli* (*p =* 0.031), enteropathogenic *E. coli* (*p =* 0.031) and Shiga-toxin-producing *E. coli* (*p =* 0.044) were recovered more commonly from cases than controls. DEC from patients with diarrhoea were commonly resistant to sulphonamides (91.3%), trimethoprim (82.6%), and ampicillin (78.3%) but were largely susceptible to quinolones and carbapenems (97.1%).

**Conclusion:**

Enteropathogenic, enterotoxigenic and Shiga toxin-producing *E. coli* are associated with diarrhoea in our setting, and show considerable resistance to first-line antimicrobials. Risk factors for DEC diarrhoea include infancy, inadequate breastfeeding and caregivers with education below primary school.

**Supplementary Information:**

The online version contains supplementary material available at 10.1186/s12879-024-09793-0.

## Introduction

Globally, diarrhoeal diseases are the second biggest cause of mortality for children under the age of five, accounting for one in nine child fatalities [1]. In resource-limited countries, Nigeria inclusive, diarrhoeal diseases are a major cause of morbidity [2–4] and diarrhoeagenic *Escherichia coli* (DEC) make an important but uncommonly qualified contribution to the problem [5]. The epidemiological significance of different DEC categories in childhood diarrhoea varies from one geographical area to another, and there are important regional differences in the prevalence of the different categories of DEC over time and seasons. In spite of these differences, few studies have been carried out in Africa to investigate the burden of the pathogens [4, 6–8].

## Materials and methods

### Study population

A total of 501 children aged between 0 and 60 months were recruited between October, 2016 and October, 2017 from primary, secondary, and tertiary hospitals (Enuwa Primary Health Center, Oke-Ogbo State Hospital Ile-Ife, Wesley Guild Hospital Ilesa, and Obafemi Awolowo University Teaching Hospitals Complex Ile-Ife) in Osun State, Nigeria, with a case and control proportion ratio of 1:2. This was calculated from Kelsey et al. [9] formula; $n=\left(\frac{r+1}{r}\right) \frac{(\bar{p})(1-\bar{p})\left(Z_\beta+Z_{\alpha / 2}\right)^2}{\left(p_1-p_2\right)^2}${where n= Sample size, Z_α/2_=0.84 (for 80% power), Z_β_=1.96 (for 0.05 significance level), *r*= 2, p_1_= proportion exposed in the control group 24.9% Onanuga et al. [6], p_2_= Prevalence of DEC 37.1% Okeke et al. [7], with a 10% attrition rate}. Diarrhoea cases were children less than 5 years old that experienced three or more loose stools within 24-hours, while controls were children without diarrhoea visiting the same healthcare facility during the study period.

### Specimen collection and processing

Using clean, sterile, and leak-proof universal bottles, fresh stool samples were collected from diarrhoea children (under five-year-olds) as well as age-matched, apparently healthy counterparts. All fresh stool samples of participants were inoculated into Selenite F broth, and on Eosin Methylene Blue agar and MacConkey agar plates (Oxoid Ltd., Hampshire, England) and incubated aerobically for 24 h at 37ºC. Following aseptic sub-culturing from Selenite F broth, plates of Salmonella Shigella Agar (SSA) were incubated aerobically at 37^º^C for 24 h. Up to five distinct lactose-fermenting colonies were aseptically picked from Eosin Methylene Blue agar and MacConkey agar plates, streaked on Nutrient Agar (Oxoid Ltd., Basingstoke, Hampshire, England) and incubated aerobically at 37^º^C for 24 h [10] and archived in a glycerol broth and stored in a freezer at -80°. The isolates were identified by conventional tube biochemical tests and confirmed with Microbact™ 24E identification kit (Oxoid Ltd., Basingstoke Hampshire, England). *E. coli* ATCC 25,922 served as the control strain.

### Antimicrobial susceptibility testing

Antibiotic susceptibility testing against thirteen antimicrobials was performed using the modified Kirby-Bauer disc diffusion technique, following the guidelines set by the Clinical Laboratory Standards Institute [11]. The discs tested were ampicillin (10 µg), streptomycin (10 µg), trimethoprim (5 µg), tetracycline (30 µg), Nalidixic acid (30 µg), chloramphenicol (30 µg), ciprofloxacin (5 µg), sulphonamide (300 µg), cefotaxime (30 µg), ceftazidime (30 µg), cefoxitin (30 µg), amoxicillin-clavulanate (20/10µg) and ertapenem (10 µg) (Oxoid Ltd., Basingstoke Hampshire, England). *E. coli* ATCC 25,922 served as the control strain.

### Detection of diarrhoeagenic *E. coli* virulence gene

DNA extraction was done using the Wizard Genomic DNA extraction Kit (Promega Corporation, Madison, USA) in accordance to manufacturer’s protocol [12] using aseptic precautions. A multiplex polymerase chain reaction (PCR) technique was used to screen all *Escherichia coli* for specific diarrhoea determinant genes [13]. The Multiplex PCR was grouped into PCR 1, PCR 2 and PCR 3 [13, 14]. Multiplex PCR assay 1 utilised three primer pairs and detected the presence of *eae* (Typical EPEC, atypical EPEC and EHEC are positive), *bfpA* (Only typical EPEC are positive, and target of *CVD432* (typical EAEC are positive). Detection of *ETEC*,* EHEC*,* STEC*,* EIEC* and *Shige* PCR assay 2 used five primer pairs and detected the presence of LT and ST genes (ETEC are positive for one or both), *stx1*,* stx2* (EHEC, STEC and *Shigella dysentriae* type 1 are positive for one or both), and *ipaH* (*Shigella* and EIEC are positive), generating PCR products of distinct sizes which were easily distinguished after agarose gel electrophoresis as presented in Table 1 [13]. PCR2 by Aranda et al. [13] seeks five targets. Two of these targets (180 bp) and (190 bp) are very close in size. To overcome the potential of failing to discriminate them in some reactions, in our laboratory, we have separated PCR2 into two reactions leaving us with PCR 3 [13], to detect *ipaH* (EIEC and *Shigella*), LT and ST (ETEC); PCR4 detects shiga-toxin 1 and 2 (STEC and EHEC).

**Table 1:** List of PCR primers and positive controls used [13, 14]

DEC	Primer designation	Primer (5´ to 3´)	Target gene or probe	Amplicon size (bp)	Positive Control
EPEC	*eae1*	CTGAACGGCGATTACGCGAA	*eae*	917	E2348/69
	*eae2*	CCAGACGATACGATCCAG			
	*bfp1*	AATGGTGCTTGCGCTTGCTGC	*bfpA*	326	E2348/69
	*bfp2*	GCCGCTTTATCCAACCTGGTA			
EAEC	*CVD432-1*	CTGGCGAAAGACTGTATCAT	*CVD432*	630	17 − 2
	*CVD432-2*	CAATGTATAGAAATCCGCTGTT			
ETEC	*LT f*	GGCGACAGATTATACCGTGC	LT	450	H10407
	*LT r*	CGGTCTCTATATTCCCTGTT			
	*ST f*	ATTTTTMTTTCTGTATTRTCTT	ST	190	H10407
	*ST r*	CACCCGGTACARGCAGGATT			
EIEC	*ipaH1*	GTTCCTTGACCGCCTTTCCGATACCGTC	*ipaH*	600	E137
	*ipaH2*	GCCGGTCAGCCACCCTCTGAGAGTAC			
STEC/EHEC	*stx1f*	ATAAATCGCCATTCGTTGACTAC	*stx*1	244	EDL933
	*stx1r*	AGAACGCCCACTGAGATCATC			
	*stx2f*	GGCACTGTCTGAAACTGCTCC	*Stx2*	190	EDL933
	*stx2r*	TCGCCAGTTATCTGACATTCTG			

EPEC- enteropathogenic *E. coli* EHEC-enterohaemorrhagic *E. coli*; EAEC-enteroaggregative *E. coli* ETEC- enterotoxigenic *E. coli*; EIEC – enteroinvasive *E. coli STEC-*shiga-toxin-producing *E-coli*

### Data analysis

Bivariate analysis of categorical variables was conducted using the Chi-Square test in Epi Info™ to evaluate the association between variables. When the expected frequency in any category was less than five, Fisher’s exact test was applied. Inferences were made based on the computed percentage positivity, 95% confidence intervals, and *p*-values. The level of significance was set at *p*˂ 0.05.

## Results

A total of 501 children under the age of five were recruited over the study period (167 cases and 334 controls). Among the diarrhoea cases; there were 38 (22.8%) infants (less than 12 months), with 82 (49.1%) females and 85 (50.9%) males. As presented in Table 2, age < 12 months (38 [49%]), ϰ²(1) = 9.839, *p* = 0.002); caregivers with education below primary school (40 [46%]), ϰ²(1) = 7.574, *p* = 0.006); duration of breastfeeding < 6 months (21 [75%]), ϰ²(1) = 23.170, *p* < 0.001); and caregivers who were siblings (160 [78%]), ϰ²(1) = 8.146, *p* = 0.004), were significantly associated with diarrhoea.

**Table 2:** Demographic characteristics and risk factors of the samples used in the study

Socio-demographic parameters	Cases (*N* = 167) *n*(%)	Controls (*N* = 334) *n*(%)	Total (*N* = 501) *n*(%)	χ^2^ (df)	*p*-value
GENDER				1.603 (1)	0.205
Male	85 (50.90)	150 (63.82)	235 (46.91)		
Female	82 (30.83)	184 (69.17)	266 (53.09)		
AGE (in months)				9.839 (1)	0.002*
˂ 12 months	38 (48.72)	40 (51.28)	78 (15.57)		
˃ 12 months	129 (30.50)	294 (69.50)	423 (84.43)		
EDUCATION				7.574 (1)	0.006*
Primary	40 (45.98)	47 (54.02)	87 (17.37)		
Secondary & Tertiary	127 (30.67)	287 (69.32)	414 (82.63)		
WATER SOURCE				2.318 (1)	0.128
Well	83 (30.40)	190 (69.60)	273 (54.49)		
Tap/Treated water	84 (36.84)	144 (63.15)	228 (45.51)		
BREAST FEEDING DURATION				23.170 (1)	<0.001*
6 months.	21 (12.60)	7 (25.00)	28 (5.59)		
6 months	146 (30.87)	327 (69.13)	473 (94.41)		
CARE-GIVER				8.146 (1)	0.004*
Mother	160 (32.52)	332 (67.47)	492 (98.20)		
Sibling	7 (77.78)	2 (22.22)	9 (1.80)		
Total	167 (100.00)	334 (100.00)	501(100.00)		

*N* = 501; *p < 0.05 (i.e. Statistically Significant); ϰ^2^= Chi square

Despite enrichment, *Salmonella* spp. was not recovered from any specimen in the study. A total of 618 *E. coli* isolates were obtained from the specimens. Out of these, PCR identified 256 as DEC, which originated from 155 unique individuals: 69 from cases and 86 from controls. DEC was recovered significantly more in cases, (69 [41.3%]), ϰ²(1) = 12.630, *p* < 0.001). As shown in Table 3, the most commonly detected DEC pathotype was enterotoxigenic *E. coli* (ETEC), which was recovered from 49 cases and 69 controls. Most of these ETEC strains 77 (65.3%) harboured the *ST* gene, encoding heat stable enterotoxin. Enteropathogenic *E. coli* isolates were recovered from 22 individuals and seven of these instances were typical EPEC, carrying the *bfpA* gene. All the specific DEC pathotypes sought, except EAEC and EIEC occurred significantly more in cases than controls: ETEC (49 [41.5%]), ϰ²(1) = 4.662, *p* = 0.031); EPEC (12 [54.5%]), ϰ²(1) = 4.659, *p* = 0.031); and STEC (4 [80.0%]), FE = 0.044, *p* = 0.044), as presented in Table 3.

**Table 3:** Occurrence of pathotypes of DEC in cases and controls

DEC	Target gene	No (%) of Participants		Statistical test (df)	*p*- value
		Cases (*N*=167) *n* (%)	Controls (*N*=334) *n* (%)		
ETEC		**49 (41.5)**	**69 (58.5)**	ϰ^2^(1)=4.662	0.031*
	*LT*	7 (36.8)	12 (63.1)		
	*ST*	36 (46.8)	41 (53.3)		
	*LT* & *ST*	6 (22.3)	16 (72.7)		
EIEC (*ipaH*)		**4 (50.0)**	**4 (50.0)**	FE=0.257	0.450
EPEC		**12 (54.5)**	**10 (45.5)**	ϰ^2^(1)=4.659	0.031*
	*bfp+*	3 (42.9)	4 (57.1)		
	*bfp-*	9 (60.0)	6 (40.0)		
EAEC (CVD432)		**0 (0.0)**	**2 (100.0)**	FE=0.444	0.554
STEC		4 (80.0)	**1 (20.0)**	FE=0.044	0.044*
	*stx1*	2 (66.7)	1 (33.3)		
	*stx2*	1 (100.0)	0 (0.0)		
	*stx1 *& *stx2*	1 (100.0)	0 (0.0)		
Total DEC		**69 (44.5)**	**86 (55.5)**	ϰ^2^(1)=12.630	<0.001*

*p<0.05 (i.e. Statistically Significant; FE= Fisher’s Exact

EPEC- enteropathogenic *E. coli*; EAEC-enteroaggregative *E. coli*; EIEC – enteroinvasive *E. coli*; EHEC-enterohaemorrhagic *E. coli*; ETEC- enterotoxigenic *E. coli*; *STEC-*shiga-toxin-producing *E-coli*

**Fig. 1:**
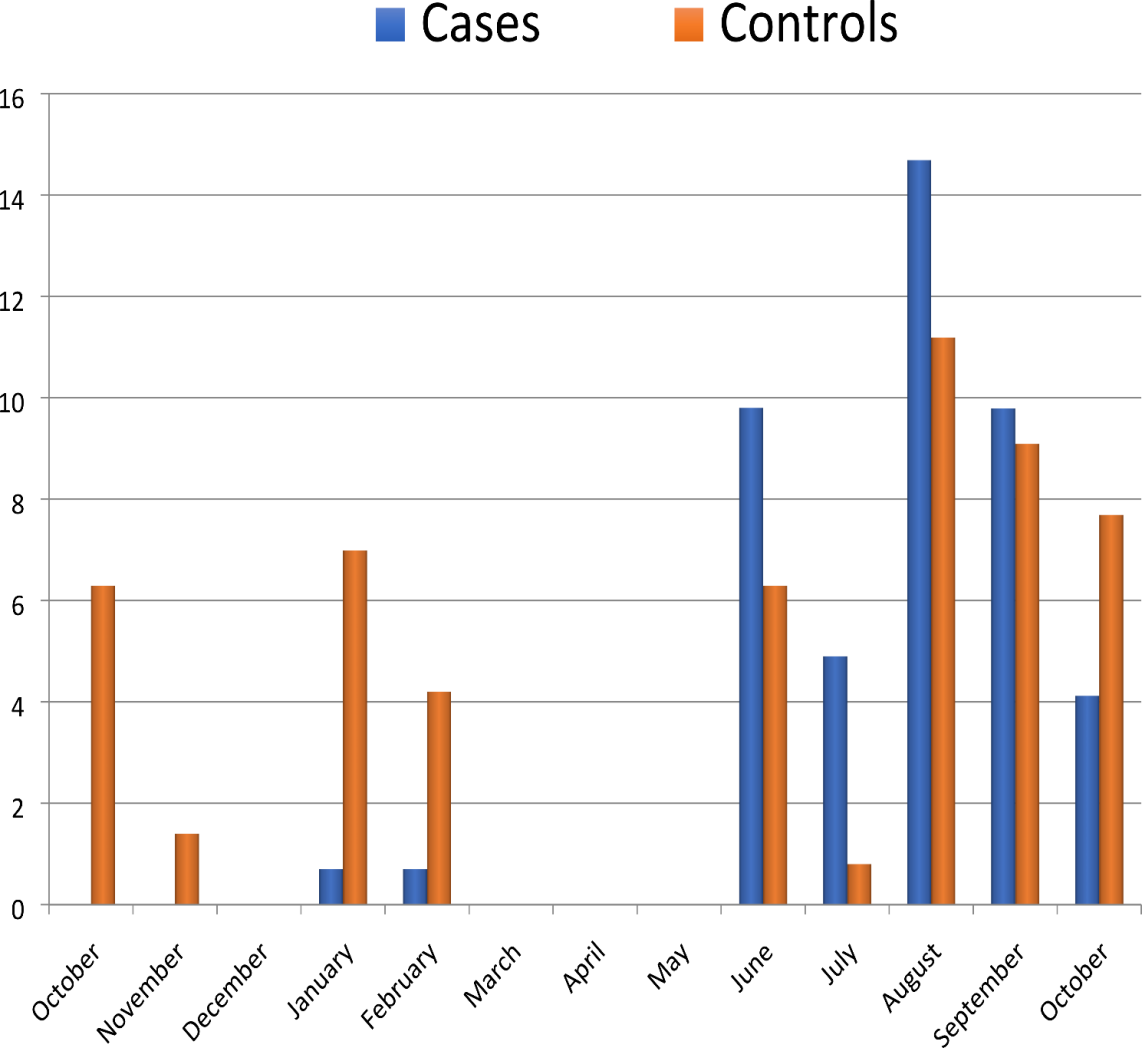
Seasonal variations of DEC in cases and controls in the study year

Seasonal variation was observed in the occurrence of DEC in terms of number and pathotypes. The rate of recovery of DEC strains from children with diarrhoea was significantly higher in the wet season (March to October) (65 [94.2%]) in contrast to the dry season (4; 5.8%) (*p <* 0.001) as presented in Fig. 1.

Table 4 shows that among the diarrhoeal cases, there was high rates of resistance of the isolated DEC to commonly prescribed antibiotics, namely sulphonamide (63; 91.3%), trimethoprim (57; 82.6%), ampicillin (54; 78.3%) and tetracycline (40; 58.0%). By contrast, resistance to quinolones and carbapenems was uncommon (2.9%).

**Table 4:** Antimicrobial Resistance Profile of DEC samples

Antibiotics	EIEC (*N* = 8)		ETEC (*N* = 118)		EPEC (*N* = 22)		EAEC (*N* = 2)	STEC (*N* = 5)		TOTAL DEC(*N* = 155)		*p*-value
	Case *n* = 4(%)	Control *n* = 4(%)	Case *n* = 49(%)	Control *n* = 69(%)	Case *n* = 12(%)	Control *n* = 10(%)	Control *n* = 2(%)	Case *n* = 4(%)	Control *n* = 1(%)	Case *n* = 69(%)	Control *n* = 86(%)	
Sulphonamide	2(50.0)	1(25.0)	45(91.8)	36(52.2)	12(100.0)	0(0.0)	2(100.0)	4(100.0)	1(100.0)	63(91.3)	40(46.5)	**< 0.001**
Trimethoprim	0(0.0)	1(25.0)	42(85.7)	8(11.6)	11(91.7)	0(0.0)	1(50.0)	4(100.0)	1(100.0)	57(82.6)	11(12.8)	**< 0.001**
Ampicillin	0(0.0)	1(25.0)	40(81.6)	26(52.2)	10(83.3)	8(80.0)	1(50.0)	4(100.0)	0(0.0)	54(78.3)	35(40.7)	**< 0.001**
Tetracycline	0(0.0)	0(0.0)	29(59.2)	21(30.4)	8(66.7)	0(0.0)	1(50.0)	3(75.0)	1(100.0)	40(57.9)	21(24.4)	**< 0.001**
Amoxicillin-clavulanic acid	0(0.0)	1(25.0)	18(36.7)	15(21.7)	9(75.0)	8(80.0)	0(0.0)	3(75.0)	1(100.0)	30(43.5)	25(29.1)	0.007
Streptomycin	0(0.0)	0(0.0)	12(24.5)	13(18.8)	5(41.7)	0(0.0)	0(0.0)	3(75.0)	0(0.0)	20(28.9)	14(11.6)	0.078
Cefotaxime	0(0.0)	1(25.0)	10(20.4)	7(10.1)	6(50.0)	5(50.0)	1(50.0)	3(75.0)	0(0.0)	20(28.9)	14(11.6)	0.078
Ceftazidime	0(0.0)	1(25.0)	9(18.4)	6(8.7)	4(33.3)	3(30.0)	0(0.0)	1(25.0)	0(0.0)	14(20.3)	10(11.6)	0.181
Cefoxitin	0(0.0)	1(25.0)	4(8.1)	6(8.7)	4(41.7)	1(10.0)	0(0.0)	1(25.0)	0(0.0)	10(21.7)	8(3.4)	0.327
Chloramphenicol	0(0.0)	0(0.0)	3(6.1)	2(2.9)	0(0.0)	0(0.0)	0(0.0)	0(0.0)	0(0.0)	3(4.3)	2(2.3)	0.656
Ertapenem	0(0.0)	0(0.0)	2(4.1)	2(2.9)	0(0.0)	0(0.0)	0(0.0)	0(0.0)	0(0.0)	2(2.9)	2(2.3)	1.000
Nalidixic acid	0(0.0)	0(0.0)	2(4.1)	1(1.4)	1(8.3)	1(10.0)	0(0.0)	0(0.0)	0(0.0)	2(2.9)	1(1.2)	0.586
Ciprofloxacin	0(0.0)	0(0.0)	0(0.0)	0(0.0)	0(0.0)	0(0.0)	1(50.0)	2(50.0)	0(0.0)	2(2.9)	1(1.2)	0.586

*EAEC cases = 0

## Discussion

Diarrhoeagenic *Escherichia coli* strains are pathogens of great public health importance, affecting both adults and children worldwide, but are infrequently sought because molecular or tissue culture methods are required to delineate them from commensals [6, 14]. While rotavirus often takes center stage as a leading cause of childhood diarrhoea, particularly in Africa, DEC strains, especially ETEC, EAEC, and EPEC, can contribute significantly to the overall burden [5, 14, 15]. The epidemiological significance of each DEC pathotypes in childhood diarrhoea varies from one geographical area to another [5]. Also, there are important regional differences in the prevalence of the different categories of DEC over time and seasons [5, 8, 14–18]. The prevalence of three DEC categories (ETEC, EAEC, and EPEC), was significantly higher in cases than in the controls (p˂0 001). Similar outcomes have also been reported in western (Ghana and Nigeria) [6, 19, 20] and south-eastern Africa (Mozambique) [21]. The fact that DEC was recovered from 23.7% of the controls in this study area shows that healthy children, who might act as reservoirs for transmission and/or suffer long term consequences of colonization [22], also harbour these pathogens. In this study, the five common pathotypes of *E. coli*—ETEC, EIEC, EPEC, EAEC, and STEC were identified; where ETEC, EPEC and STEC were significantly recovered more in diarrhoea cases than controls. This is consistent with the findings of other researchers from other developing countries, where the frequencies of recovery of ETEC [21, 23–25], EPEC [21], and STEC [23] were significantly higher in the cases than in the controls. Enterotoxigenic *Escherichia coli* (ETEC)-associated diarrhoea has been reported by many studies as the most common bacterial diarrhoea affecting children under 5 years old living in developing countries, as well as travellers to these countries [26]. In this study, ETEC was the most prevalent DEC pathotype, among both cases and controls, and was significantly associated with diarrhoea. This figure was comparable to findings from many [23, 27–29], but not all [6, 30, 31] other resource-limited countries. In addition, in a study on DEC among children with and without diarrhoea in Burkina Faso by Bonkoungou et al., ETEC was highly significantly associated with diarrhoea [32]. Altogether, reviews on DEC in sub-Saharan Africa stated that ETEC is associated with infantile diarrhoea in African countries and also the most common cause of acute diarrhoea [5, 33].

As in other studies, DEC were most predominantly recovered from children under one year of age [14, 8, 20, 25, 34, 35]. This age group represents a particularly vulnerable population to DEC infections due to factors such as immature immune systems and increased susceptibility to environmental pathogens [7, 14, 36]. Additionally, our study highlighted the significance of inadequate breastfeeding (less than 6 months) as another notable risk factor for DEC infection. This is comparable to what Ali et al. [37] and Akinlabi et al. [38] observed in northern and southwestern Nigeria. Breastfeeding provides infants with essential nutrients and antibodies that bolster their immune defences against infections, including DEC-related ones [14, 36–38]. Therefore, the absence or early cessation of breastfeeding may leave infants more susceptible to diarrhoeal illnesses, including those caused by DEC. Our finding and similar reports from elsewhere emphasize the importance of promoting exclusive breastfeeding for the first six months of life as an infectious diarrhoea prevention measure [38, 39].

Our study revealed a significant association between caregivers with a limited educational background and DEC infection. This finding underscores the multifactorial nature of diarrhoeal illnesses in children, highlighting the impact of socioeconomic factors on disease transmission [39, 40]. Caregivers with lower levels of education may have limited access to health education and resources, leading to suboptimal hygiene practices and increased susceptibility to DEC contamination in the household environment [39–41]. Our study also identified caregivers who are siblings as being associated with DEC infection among children. This suggests the potential contribution of intra-familial transmission routes in DEC spread. Siblings may facilitate close contact and shared exposure to contaminated environments, thereby increasing the risk of transmission within households [42].

This study revealed a seasonal variation in the prevalence of DEC infection in the environment. When compared to the dry season, the overall prevalence of DEC was shown to be significantly higher in the rainy season (*p <* 0.001). The peak prevalence of DEC (ETEC and EPEC) was observed in August, which is regarded as one of the months with the most rainfall in the study area, and is characterized by the contamination of surface waterways by sewage spills, faeces spills, and other waste spills. This finding is consistent with earlier research on the seasonal variation of DEC infection, including those by Onanuga et al., Tumwine et al., and El Metwally et al. [6, 43, 44].

Antimicrobial drug resistance in bacteria that cause diarrhoeal disease is a serious and growing problem [45]. Antimicrobials are not indicated for the treatment of most childhood diarrhoea diseases [46] but should be administered to children with invasive or protracted infections. Moreover, resistance in enteric isolates provides a picture of the gut reservoir of resistance genes, which can be transmitted to enteric organisms causing infections in other niches [14].

This study revealed high rates of antibiotic resistance among different DEC categories, in particular, resistance to sulphonamide, trimethoprim, amoxicillin clavulanic-acid, streptomycin, ampicillin and tetracycline, probably as a result of its increased availability of multiple generic formulations in the markets [8, 47] but also because mobile genes encoding resistance to these antimicrobials are often linked and transmitted together [14]. Substandard oral drugs, which are common in our setting [6, 14], often fail to be fully absorbed, leaving behind enough drug in the intestine to select for resistant enteric bacteria [48, 49]. Notably, the DEC isolates in this study remained susceptible to quinolones and carbapenems, highlighting the importance of preserving these critically-important antibiotics for severe infections [50]. *Salmonella* spp. was not recovered in this study despite enrichment and have been found uncommon in childhood diarrhoea studies performed in or close to our study area [6, 14, 38, 51]. As with some of those studies, we additionally did not identify any *Shigella* or many EIEC.

This study has several limitations. To begin with, controls were not time-matched with cases, possibly introducing temporal confounding. Secondly, only 1–2 isolates per specimen were screened for DEC by PCR. This limited screening might have affected our results, as typically screening 3–5 colonies can increase the yield of DEC, especially in children without diarrhoea who often carry multiple *E. coli* lineages. Akinlabi et al. [51] recently reported that the Aranda et al. [13] multiplex PCR protocol, which they compared with whole genome sequencing, had suboptimal sensitivity and specificity for certain DEC lineages that are common elsewhere in south western Nigeria. As we did not also identify DEC by whole genome sequencing, the degree to which this might affect our results is unclear. These limitations notwithstanding, the data point to a high incidence of DEC infections and a strong association with diarrhoea for multiple pathotypes. This highlights the need for more in-depth investigations using predictive tools in this study area, as well as further study of the isolates we obtained.

## Conclusion

This study shows that DEC, particularly ETEC, EPEC, and STEC are important diarrhoeagenic agents in Ile-Ife and Ilesa, Nigeria. These findings highlight the need for routine surveillance and focused tool development against diarrhoeal disease. The prevalence of ETEC and EPEC, especially during the wet season, underscores the need for routine surveillance and targeted interventions to curb their spread. Notably, the study identified crucial risk factors like infancy, inadequate breast feeding and caregivers with education below primary school, emphasizing the importance of strengthening public health programs that promote optimal infant feeding practices, hygiene education, and safe water access. Furthermore, the substantial antibiotic resistance observed among DEC isolates necessitates the judicious use of antimicrobials and the exploration of alternative treatment strategies. By delving deeper into the specific virulence factors of prevalent DEC pathotypes and their environmental interactions, future research can pave the way for the development of effective diagnostics, and targeted interventions. This study not only contributes valuable knowledge to the fight against childhood diarrhoeal disease in Nigeria but also serves as a springboard for further research efforts aimed at protecting the health and well-being of vulnerable children, particularly those under five years of age, globally.

## Electronic supplementary material

Below is the link to the electronic supplementary material.

Supplementary Material 1

## Data Availability

The datasets used during the current study are available from the corresponding author (olayinkaademolaa@gmail.com) upon reasonable request.

## Abbreviations

*E. coli*: *Escherichia coli*
DEC: Diarrhoeagenic *E. coli*
CLSI: Clinical Laboratory Standard Institute
EPEC: Enteropathogenic *Escherichia coli*
ETEC: Enterotoxigenic *Escherichia coli*
EIEC: Enteroinvasive *Escherichia coli*
EAEC: Enteroaggregative *Escherichia coli*
EHEC / STEC: Enterohemorrhagic (Shiga toxin- producing) *Escherichia coli*
DAEC: Diffusely adherent *Escherichia coli*
STEC: Shiga toxin producing *E. coli*
