# Supplementary material for: Diarrhoeagenic Escherichia coli associated with childhood diarrhoea in Osun state, Nigeria
Source: BMC Infect Dis. 2024 Sep 8;24:928. doi: 10.1186/s12879-024-09793-0 (PMC11382472; doi:10.1186/s12879-024-09793-0)
Supplement: Supplementary file 1 — Supplementary Material 1 [file 12879_2024_9793_MOESM1_ESM.docx]

**Appendix-I**

**Title of the research:** Pathogenic lineages of enteric bacteria in Nigeria

**Name(s) and affiliation(s) of researcher(s) of applicant(s):** This study is being conducted by Professor Iruka N. Okeke of the University of Ibadan, and Prof. Oladipo A. Aboderin of Obafemi Awolowo University.

**QUESTIONNAIRE (A)**

***DIARRHOEA PATIENT***

1.0 Date: _______________ 1.1 Project Site: ______________________

1.2. Time____________

**2.0 Background of participant**

2.1 Participant code (Participant receives code after providing informed consent): ______________

2.2 Age: ____ 2.3 Sex: ____ 2.4 Area/ Residence (Town/village): ______________

2.5. Number of individuals in the child’s household__________

2.6. Number of rooms in home (all rooms, not just sleeping rooms): ___________

2.7 Who takes care of child most of the time especially week days? ___________

2.8 Weight of child: _____________________

2.9 Height of child: _____________________

**3.0 History of diarrhoea**

3.1 Frequency in a day: __________ 3.2 For how long? _________________

3.3 Blood in stool Yes [ ] No [ ] ___________ 3.4. Mucus in stool Yes [ ] No [ ]

3.5 Fever? Yes [ ] No [ ] Temperature ___________°C

3.6 Did you take any antibiotic/drug/medicine/herbal before visiting this clinic? Yes [ ] No [ ]

3.7 Antibiotic/drug name _________________________________________________

**4.0 How long has this child been exclusively breast feed?**

4.1. 6 months [ ]

4.2 < 6 months [ ]

4.3 others? (please state) _____________________________________

4.4 In your estimation, what is the likely cause of the diarrhoea? __________________________________________________________________

**5.0 Source of household/drinking water** [Please tick]

5.1 Tap Household [ ] ; Drinking [ ]

5.2 Tank filled from tap water Household [ ] ; Drinking [ ]

5.3 Tank filled by external provider Household [ ] ; Drinking [ ]

5.4 Well Household [ ] ; Drinking [ ]

5.5 Borehole Household [ ] ; Drinking [ ]

5.6 River or stream Household [ ] ; Drinking [ ]

5.7 Bottled Household [ ] ; Drinking [ ]

5.8 Boiled Household [ ] ; Drinking [ ]

5.9 Others (please state) _________________________________________

**6.0 Educational background of mother/ guardian** [Please tick]

6.1 Up to primary school [ ]

6.2 Up to JSS [ ]

6.3 Up to SSS/Sec. sch. [ ]

6.4 Up to Univ/Poly tech. [ ]

6.5 Others (please state) _________________________________________

**SPECIMENS (Yes/No):** CHILD Stool[Y/N] Anal Swab[Y/N];

MOTHER/CAREGIVER Stool[Y/N]

Re-enter patient Code _______________________________Check code on specimen bottles.

**Title of the research:** Pathogenic lineages of enteric bacteria in Nigeria

**Name(s) and affiliation(s) of researcher(s) of applicant(s):** This study is being conducted by Professor Iruka N. Okeke of the University of Ibadan, and Professor Oladipo A. Aboderin of Obafemi Awolowo University.

**QUESTIONNAIRE (B)**

***CONTROL FOR DIARRHOEA STUDY***

1.0 Date: _______________ 1.1 Project Site: ______________________ 1.2. Time____________

**2.0 Background of participant**

2.1 Participant code (Participant receives code after providing informed consent):______________

2.2 Age: ____ 2.3 Sex: ____ 2.4 Area/ Residence (Town/village):______________

2.5. Number of individuals in the child’s household__________

2.6. Number of rooms in home (all rooms, not just sleeping rooms)___________

2.7 Who takes care of child most of the time especially **week days**? ___________

2.8 Weight of child: _____________________

2.9 Height of child: _____________________

3.0 **How long did you exclusively breast feed?**

3.1. 6 months [ ]

3.2 < 6 months [ ]

3.3 Others (please state)_____________________________________________

**5.0 Educational background of mother/ guardian** [Please tick]

5.1 Up to primary school [ ]

5.2 Up to JSS [ ]

5.3 Up to SSS/Sec. sch. [ ]

5.4 Up to Univ/Poly tech. [ ]

5.5 Others (please state) _______________________________________________

**SPECIMENS (Yes/No):** CHILD Stool[Y/N] Anal Swab[Y/N];

MOTHER/CAREGIVER Stool[Y/N]

Re-enter patient Code _______________________________Check code on specimen bottles.
